# Development and validation of an early warning tool for sepsis and decompensation in children during emergency department triage

**DOI:** 10.1038/s41598-021-87595-z

**Published:** 2021-04-21

**Authors:** Louis Ehwerhemuepha, Theodore Heyming, Rachel Marano, Mary Jane Piroutek, Antonio C. Arrieta, Kent Lee, Jennifer Hayes, James Cappon, Kamila Hoenk, William Feaster

**Affiliations:** Children’s Health of Orange County, 1201 W La Veta Ave, Orange, CA 92868 USA

**Keywords:** Health care, Medical research, Risk factors, Diseases, Infectious diseases

## Abstract

This study was designed to develop and validate an early warning system for sepsis based on a predictive model of critical decompensation. Data from the electronic medical records for 537,837 visits to a pediatric Emergency Department (ED) from March 2013 to December 2019 were collected. A multiclass stochastic gradient boosting model was built to identify early warning signs associated with death, severe sepsis, non-severe sepsis, and bacteremia. Model features included triage vital signs, previous diagnoses, medications, and healthcare utilizations within 6 months of the index ED visit. There were 483 patients who had severe sepsis and/or died, 1102 had non-severe sepsis, 1103 had positive bacteremia tests, and the remaining had none of the events. The most important predictors were age, heart rate, length of stay of previous hospitalizations, temperature, systolic blood pressure, and prior sepsis. The one-versus-all area under the receiver operator characteristic curve (AUROC) were 0.979 (0.967, 0.991), 0.990 (0.985, 0.995), 0.976 (0.972, 0.981), and 0.968 (0.962, 0.974) for death, severe sepsis, non-severe sepsis, and bacteremia without sepsis respectively. The multi-class macro average AUROC and area under the precision recall curve were 0.977 and 0.316 respectively. The study findings were used to develop an automated early warning decision tool for sepsis. Implementation of this model in pediatric EDs will allow sepsis-related critical decompensation to be predicted accurately after a few seconds of triage.

## Introduction

In the United States, hospitalizations for severe sepsis doubled between 2000 and 2008, with an overall annual healthcare cost of $146 billion^1^. In children, sepsis is associated with high morbidity and mortality, especially among vulnerable patients with chronic conditions, who often require intensive treatment to avoid preventable death^2^. Even the limited number of pediatric deaths caused by sepsis is understood as too heavy a loss to bear when one considers that early interventions could have prevented it. While preventable deaths are rare in pediatrics, the prevalence of severe sepsis in children is increasing across the globe because of the increasing prevalence of drug-resistant infections^3^. The earliest opportunity for intervention prior to critical decompensation often arises in the emergency department (ED), the first point of contact between many patients and the healthcare system. Despite a recent increase in clinicians’ awareness of critical decompensation and severe sepsis, as well as associated changes in the corresponding diagnostic criteria^1–4^, pediatric-specific tools remain poorly developed. Stratifying risk with the systemic inflammatory response syndrome (SIRS) criteria or quick sequential [sepsis-related] organ failure assessment (qSOFA) typically takes approximately 1 h^5^. Methods that facilitate early warning and decision making may lead to process improvements and associated reductions in morbidity and mortality^6,7^. A predictive model for predicting patient death is ideal for capturing critical decompensation because even false-positive predictions help to identify patients in need of critical attention. In this study, we used stochastic gradient boosting to develop a predictive model for critical decompensation in pediatric ED patients. Stochastic gradient boosting is an approach of using multiple regression tree-based models to predict an event of interest. In this case, the trees are built sequentially in such a way that each additional tree minimizes the error of the ensemble.

## Methods

Triage data on all ED encounters from March 2013 to December 2019 at a tertiary pediatric institution were retrieved. To create an early warning tool with utility for non-emergency as well as emergency healthcare providers, outcome events were measured while the patients were in the ED or after admission to the hospital, when applicable. In evaluating the diagnosis codes entered into the electronic medical record (EMR), a multiclass outcome variable was created to capture: (1) patients who expired using all-cause mortality; (2) patients who had severe sepsis; (3) patients who had non-severe sepsis; (4) patients with positive bacteremia tests but no diagnosis of sepsis; (5) patients who experienced none of the four aforementioned events. Diagnostic codes (ICD-9-CM and ICD-10-CM) were used to identify patients with sepsis which facilitated the automated identification of sepsis—refer to the Supplemental Material for a list of codes.

Standard definitions for pediatric sepsis and severe sepsis have not been developed and validated. The Sepsis-1, Sepsis-2, and Sepsis-3 guidelines are for adult medicine with pediatric modifications^8–10^. The International Pediatric Sepsis Consensus Criteria (IPSCC) modified Sepsis-2 framework for specific pediatric age groups but the framework is complex, labor intensive, has limited overlap with sepsis diagnosed at the bedside, and is yet to be validated^9–11^. Also, less than 50% of children with septic shock were identified by the Sepsis-3 in a pediatric ICU study^12^. In July 2018, The US Centers for Disease Control and Prevention (CDC) convened a working group to outline a pediatric sepsis event case definition including a road map for future work needed to refine, validate, and apply the proposed algorithm^11^. The group, however, emphasized that recommendations for pediatrics were preliminary and subject to modification due to continued refinement and testing^11^.

A recent study indicated that (in adult medicine) diagnosis codes had greater specificity in identifying sepsis than implicit methods^13^, and that there is increasing consistency in the use of diagnosis codes^14^. Specificity may be more important than sensitivity in the definition of outcome variables for predictive modeling to reduce noise and the true false positive rates from the resulting model. In this study, we used both the ICD-10-CM R-codes (together with the corresponding ICD-9-CM codes) and explicit microbiological codes for sepsis. To compensate for potentially missed cases of sepsis and severe sepsis, we included a category of the response variable on all laboratory confirmed bacteremia.

Additional ED triage data retrieved included vital signs, use of supplemental oxygen, and skin assessment (description and color). The decision on the use of these triage data was driven by emergency physician recommendation, practical consideration of the data available, data likely to be available during triage, and the quality of data available from the triage form. After careful evaluation of the patient history as captured in the EMR, additional data such as previous diagnoses, medications administered (e.g., antibiotics), and past admission to the intensive care unit (ICU) were extracted. A complete list of variables included in the model can be seen in the summary statistics in Tables 1 and 2.

**Table 1:** Summary statistics: death and severe sepsis.

	Levels	Death		Severe sepsis	
		No	Yes	No	Yes
Age (years)		6.17 (5.29)	8.02 (6.91)	6·16 (5.29)	10·60 (6.27)
Sex	Female	143,757 (53.48)	49 (53.85)	143,720 (53.47)	86 (58.11)
	Male	124,935 (46.47)	42 (46.15)	124,915 (46.48)	62 (41.89)
	Unknown	135 (0.05)	0 (0.0)	135 (0.05)	0 (0.0)
Ethnicity	Latino or Hispanic	100,823 (37.50)	45 (49.45)	100,802 (37.50)	66 (44.59)
	Not Latino or Hispanic	167,869 (62.44)	46 (50.55)	167,833 (62.44)	82 (55.41)
	Unknown	135 (0.05)	0 (0.0)	135 (0.05)	0 (0.0)
Temperature (Celsius)		37.11 (0.91)	36.69 (1.44)	37.11 (0.91)	37.60 (1.13)
Respiratory rate (breaths/min)		26.02 (8.55)	29.00 (16.42)	26.02 (8.55)	29.46 (12.41)
Heart rate (beats/min)		118.34 (31.97)	124.58 (52.50)	118.33 (31.98)	141.78 (32.37)
Diastolic blood pressure (mmHg)		69.49 (12.08)	63.90 (20.39)	69.49 (12.08)	62.01 (15.94)
Systolic blood pressure (mmHg)		111.17 (15.27)	100.79 (26.59)	111.17 (15.·28)	100.81 (18.62)
Oxygen saturation (%)		98.99 (2.42)	90.89 (16.82)	98.99 (2.44)	95.79 (7.53)
Supplemental oxygen device	No	65,971 (24.54)	47 (51.65)	65,982 (24.55)	36 (24.32)
	Yes	202,856 (75.46)	44 (48.35)	202,788 (75.45)	112 (75.68)
Nursing assessment for skin	Failed	153,451 (57.08)	86 (94·51)	153,389 (57·07)	148 (100·00)
	Passed	115,376 (42.92)	5 (5.49)	115,381 (42.93)	148 (100.00)
Normal skin color	No	7762 (2.89)	47 (51.65)	7763 (2.89)	46 (31.08)
	Yes	261,065 (97.11)	44 (48.35)	261,007 (97.11)	102 (68.92)
**6-month medication history**					
Antiviral medications	No	263,125 (97.88)	74 (81.32)	263,082 (97.88)	117 (79.05)
	Yes	5702 (2.12)	17 (18.68)	5688 (2.12)	31 (20.95)
Antineoplastic medications	No	262,432 (97.62)	55 (60.44)	262,411 (97.63)	76 (51.35)
	Yes	6395 (2.38)	36 (39.56)	6359 (2.37)	72 (48.65)
Non-topical antibiotics	No	251,771 (93.66)	45 (49.45)	251,778 (93.68)	38 (25.68)
	Yes	17,056 (6.34)	46 (50.55)	16,992 (6.32)	110 (74.32)
Carbapenems	No	264,989 (98.57)	58 (63.74)	264,986 (98.59)	61 (41.22)
	Yes	3838 (1.43)	33 (36.26)	3784 (1.41)	87 (58.78)
Immunological agents	No	261,834 (97.40)	61 (67.03)	261,803 (97.41)	92 (62.16)
	Yes	6993 (2.60)	30 (32.97)	6967 (2·59)	56 (37.84)
Topical antibiotics	No	259,551 (96.55)	65 (71.43)	259,552 (96.57)	64 (43.24)
	Yes	9276 (3.45)	26 (28.57)	9218 (3.43)	84 (56.76)
Viral vaccines	No	209,630 (77.98)	75 (82.42)	209,634 (78.00)	71 (47.97)
	Yes	59,197 (22.02)	16 (17.58)	59,136 (22.00)	77 (52.03)
Intravenous medications	No	169,591 (63.09)	7 (7.69)	169,598 (63.10)	148 (100.00)
	Yes	99,236 (36.91)	84 (92.31)	99,172 (36.90)	148 (100.00)
Use of gastrointestinal tubes	No	262,410 (97.61)	57 (62.64)	262,383 (97.62)	84 (56.76)
	Yes	6417 (2.39)	34 (37.36)	6387 (2.38)	64 (43.24)
**6-month healthcare utilization and diagnoses history**					
New patients (no historical data)		77,640 (28.92)	13 (14.29)	77,634 (28.91)	19 (12.84)
Previous ICU admission	No	183,458 (68.24)	23 (25.27)	183,423 (68.25)	58 (39.19)
	Yes	7638 (2.84)	55 (60.44)	7622 (2·84)	71 (47.97)
Max. previous length of stay	-	6.46 (11.33)	20.33 (21.10)	6.45 (11.33)	18.48 (17.81)
Previous ED visits	No	32,356 (12.04)	44 (48.35)	32,324 (12.03)	76 (51.35)
	Yes	158,740 (59.05)	34 (37.36)	158,721 (59.05)	53 (35.81)
Previous outpatient encounters	No	133,068 (49.50)	17 (18.68)	133,063 (49.51)	22 (14.86)
	Yes	58,028 (21.59)	61 (67.03)	57,982 (21.57)	107 (72.30)
Previous diagnosis for sepsis	No	189,593 (70.53)	55 (60.44)	189,580 (70.54)	68 (45.·95)
	Yes	1503 (0.56)	23 (25.27)	1465 (0.55)	61 (41.22)
Previous diagnosis for severe sepsis	No	190,716 (70.94)	66 (72.53)	190,706 (70.96)	76 (51.35)
	Yes	380 (0.14)	12 (13.19)	339 (0.13)	53 (35·81)
Infectious and parasitic diseases (A00–B99)	No	162,470 (60.44)	35 (38.46)	162,459 (60.45)	46 (31.08)
	Yes	28,626 (10·65)	43 (47·25)	28,586 (10·64)	83 (56·08)
Malignant neoplasms (C00–C96)	No	187,548 (69·77)	53 (58·24)	187,503 (69·76)	98 (66.22)
	Yes	3548 (1·32)	25 (27·47)	3542 (1·32)	31 (20.95)
In situ, benign, and other neoplasms (D00–D49)	No	187,474 (69.74)	66 (72·53)	187,427 (69.74)	113 (76.35)
	Yes	3622 (1.35)	12 (13.19)	3618 (1·35)	16 (10.81)
Diseases of the blood and immune system (D50–D89)	No	180,312 (67.07)	31 (34.07)	180,294 (67.08)	49 (33.11)
	Yes	10,784 (4.01)	47 (51.65)	10,751 (4.00)	80 (54.05)
Endocrine, nutritional, and metabolic diseases (E00–E89)	No	170,707 (63·50)	20 (21.98)	170,682 (63.50)	45 (30.41)
	Yes	20,389 (7.58)	58 (63.74)	20,363 (7.58)	84 (56.76)
Mental, behavioral, and neurodevelopmental disorders (F01–F99)	No	169,866 (63.19)	36 (39.56)	169,842 (63.19)	60 (40.54)
	Yes	21,230 (7.90)	42 (46.15)	21,203 (7.89)	69 (46.62)
Nervous system diseases (G00–G99)	No	175,830 (65.41)	29 (31.87)	175,803 (65.41)	56 (37.84)
	Yes	15,266 (5.68)	49 (53.85)	15,242 (5.67)	73 (49.32)
Diseases of the eye and adnexa (H00–H59)	No	175,655 (65.34)	58 (63.74)	175,601 (65.34)	112 (75·68)
	Yes	15,441 (5.74)	20 (21.98)	15,444 (5.75)	17 (11.49)
Diseases of the ear and mastoid process (H60–H95)	No	168,266 (62.59)	67 (73.63)	168,221 (62.59)	112 (75.68)
	Yes	22,830 (8.49)	11 (12.09)	22,824 (8.49)	17 (11.49)
Circulatory system diseases (I00–I99)	No	182,478 (67.88)	26 (28.57)	182,440 (67.88)	64 (43.24)
	Yes	8618 (3.21)	52 (57.14)	8605 (3.20)	65 (43.92)
Respiratory diseases (J00–J99)	No	121,248 (45.10)	21 (23.08)	121,227 (45.10)	42 (28.38)
	Yes	69,848 (25.98)	57 (62.64)	69,818 (25.98)	87 (58.78)
Digestive diseases (K00–K95)	No	155,984 (58.02)	30 (32.97)	155,969 (58.03)	45 (30.41)
	Yes	35,112 (13.06)	48 (52.75)	35,076 (13.05)	84 (56.76)
Skin and subcutaneous diseases (L00–L99)	No	171,984 (63.98)	48 (52.75)	171,959 (63.98)	73 (49.32)
	Yes	19,112 (7.11)	30 (32.97)	19,086 (7.10)	56 (37.84)
Musculoskeletal and connective tissue diseases (M00–M99)	No	168,987 (62.86)	44 (48.35)	168,963 (62.87)	68 (45.95)
	Yes	22,109 (8.22)	34 (37.36)	22,082 (8.22)	61 (41.22)
Genitourinary diseases (N00–N99)	No	172,781 (64.27)	44 (48.35)	172,752 (64.28)	73 (49.32)
	Yes	18,315 (6.81)	34 (37.36)	18,293 (6.81)	56 (37.84)
Pregnancy and related conditions (O00–O9A)	No	190,565 (70.89)	78 (85.71)	190,515 (70.88)	128 (86.49)
	Yes	531 (0.20)	13 (14.29)	530 (0.20)	1 (0.68)
Conditions from perinatal period (P00–P96)	No	180,965 (67.32)	62 (68.13)	180,913 (67.31)	114 (77·03)
	Yes	10,131 (3.77)	16 (17.58)	10,132 (3.77)	15 (10.14)
Congenital malformations, deformations, and chromosomal abnormalities (Q00–Q99)	No	176,173 (65.53)	55 (60.44)	176,150 (65·54)	78 (52.70)
	Yes	14,923 (5.55)	23 (25.27)	14,895 (5.54)	51 (34.46)
Injury and poisoning (S00–T88)	No	139,709 (51.97)	35 (38.46)	139,684 (51.97)	60 (40.54)
	Yes	51,387 (19.12)	43 (47.25)	51,361 (19.11)	69 (46.62)

**Table 2:** Summary statistics: non-severe sepsis and bacteremia.

Variable	Levels	Non-severe Sepsis		Bacteremia	
		No	Yes	No	Yes
Age (years)		6.16 (5.29)	7.73 (6.70)	6.16 (5.29)	6.79 (6.21)
Sex	Female	143,515 (53.48)	291 (51.23)	143,479 (53.47)	327 (55.90)
	Male	124,700 (46.47)	277 (48.77)	124,719 (46.48)	258 (44.10)
	Unknown	135 (0.05)	0 (0.0)	135 (0.05)	0 (0.0)
Ethnicity	Latino or Hispanic	100,632 (37.50)	236 (41.55)	100,620 (37.50)	248 (42.39)
	Not Latino or Hispanic	167,583 (62.45)	332 (58.45)	167,578 (62.45)	337 (57.61)
	Unknown	135 (0.05)	0 (0.0)	135 (0.05)	0 (0.0)
Temperature (Celsius)		37.11 (0.91)	37.51 (1.08)	37.11 (0.91)	37.50 (1.90)
Respiratory rate (breaths/min)		26.01 (8.54)	32.43 (13.19)	26.02 (8.55)	30.11 (11.69)
Heart rate (beats/min)		118.28 (31.95)	147.89 (31.53)	118.28 (31.96)	145.46 (31.93)
Diastolic blood pressure (mmHg)		69.49 (12.07)	65.03 (17.91)	69.49 (12.08)	65.96 (13.24)
Systolic blood pressure (mmHg)		111.19 (15.27)	102.97 (17.34)	111.18 (15.28)	105.83 (16.00)
Oxygen saturation (%)		98.99 (2.42)	96.37 (7.32)	98.99 (2·44)	97.95 (4.36)
Supplemental oxygen device	No	65,914 (24.56)	104 (18.31)	65,964 (24.58)	54 (9.23)
	Yes	202,436 (75.44)	464 (81.69)	202,369 (75.42)	531 (90.77)
Nursing assessment for skin	Failed	153,008 (57.02)	529 (93.13)	153,020 (57.03)	517 (88.38)
	Passed	115,342 (42.98)	39 (6.87)	115,313 (42.97)	68 (11.62)
Normal skin color	No	7669 (2.86)	140 (24.65)	7702 (2.87)	107 (18.29)
	Yes	260,681 (97.14)	428 (75.35)	260,631 (97.13)	478 (81.71)
**6-month medication history**					
Antiviral medications	No	262,760 (97.92)	439 (77.29)	262,765 (97.92)	434 (74.19)
	Yes	5590 (2.08)	129 (22.71)	5568 (2.08)	151 (25.81)
Antineoplastic medications	No	262,118 (97.68)	369 (64.96)	262,112 (97.68)	375 (64.10)
	Yes	6232 (2.32)	199 (35.04)	6221 (2.32)	210 (35.90)
Non-topical antibiotics	No	251,599 (93.76)	217 (38.20)	251,629 (93.77)	187 (31.97)
	Yes	16,751 (6.24)	351 (61.80)	16,704 (6.23)	398 (68.03)
Carbapenems	No	264,703 (98.64)	344 (60.56)	264,710 (98.65)	337 (57.61)
	Yes	3647 (1.36)	224 (39.44)	3623 (1.35)	248 (42.39)
Immunological agents	No	261,471 (97.44)	424 (74.65)	261,527 (97.46)	368 (62.91)
	Yes	6879 (2.56)	144 (25.35)	6806 (2.54)	217 (37.09)
Topical antibiotics	No	259,290 (96.62)	326 (57.39)	259,281 (96.63)	335 (57.26)
	Yes	9060 (3.38)	242 (42.61)	9052 (3.37)	250 (42.74)
Viral vaccines	No	209,400 (78.03)	305 (53.70)	209,462 (78.06)	243 (41.54)
	Yes	58,950 (21.97)	263 (46.30)	58,871 (21.94)	342 (58.46)
Intravenous medications	No	169,597 (63.20)	1 (0.18)	169,597 (63.20)	1 (0.17)
	Yes	98,753 (36.80)	567 (99.82)	98,736 (36.80)	584 (99.83)
Use of gastrointestinal tubes	No	262,110 (97.67)	357 (62.85)	262,053 (97.66)	414 (70.77)
	Yes	6240 (2.33)	211 (37.15)	6280 (2.34)	171 (29.23)
**6-month healthcare utilization and diagnoses history**					
New patients (no historical data)		77,549 (28.93)	104 (18.31)	77,076 (28.94)	83 (14.18)
Previous ICU admission	No	183,225 (68.28)	256 (45.07)	183,166 (68.26)	315 (53.85)
	Yes	7485 (2.79)	208 (36.62)	7506 (2.80)	187 (31.97)
Max. previous length of stay		6.37 (11.24)	15.33 (16.86)	6.30 (11.11)	19.35 (19.11)
Previous ED visits	No	32,144 (11.98)	256 (45.07)	32,150 (11.98)	250 (42.74)
	Yes	158,566 (59.09)	208 (36.62)	158,522 (59.08)	252 (43.08)
Previous outpatient encounters	No	132,968 (49.55)	117 (20.60)	132,943 (49.54)	142 (24.27)
	Yes	57,742 (21.52)	347 (61.09)	57,729 (21.51)	360 (61.54)
Previous diagnosis for sepsis	No	189,420 (70.59)	228 (40.14)	189,281 (70.54)	367 (62.74)
	Yes	1290 (0.48)	236 (41.55)	1391 (0.52)	135 (23.08)
Previous diagnosis for severe sepsis	No	190,399 (70.95)	383 (67.43)	190,327 (70.93)	455 (77.78)
	Yes	311 (0.12)	81 (14.26)	345 (0.13)	47 (8.03)
Infectious and parasitic diseases (A00–B99)	No	162,339 (60.50)	166 (29.23)	162,268 (60.47)	237 (40.51)
	Yes	28,371 (10.57)	298 (52.46)	28,404 (10.59)	265 (45.30)
Malignant neoplasms (C00–C96)	No	187,210 (69.76)	391 (68.84)	187,249 (69.78)	352 (60.17)
	Yes	3500 (1.30)	73 (12.85)	3423 (1.28)	150 (25.64)
In situ, benign, and other neoplasms (D00–D49)	No	187,125 (69.73)	415 (73.06)	187,119 (69.73)	421 (71.97)
	Yes	3585 (1.34)	49 (8.63)	3553 (1.32)	81 (13.85)
Diseases of the blood and immune system (D50–D89)	No	180,103 (67.11)	240 (42.25)	180,129 (67.13)	214 (36.58)
	Yes	10,607 (3.95)	224 (39.44)	10,543 (3.93)	288 (49.23)
Endocrine, nutritional, and metabolic diseases (E00–E89)	No	170,527 (63·55)	200 (35·21)	170,484 (63·53)	243 (41.54)
	Yes	20,183 (7.52)	264 (46.48)	20,188 (7.52)	259 (44·27)
Mental, behavioral, and neurodevelopmental disorders (F01–F99)	No	169,629 (63.21)	273 (48.06)	169,576 (63.20)	326 (55.73)
	Yes	21,081 (7.86)	191 (33.63)	21,096 (7.86)	176 (30.09)
Nervous system diseases (G00–G99)	No	175,594 (65.43)	265 (46.65)	175,497 (65.40)	362 (61.88)
	Yes	15,116 (5.63)	199 (35.04)	15,175 (5.66)	140 (23.93)
Diseases of the eye and adnexa (H00–H59)	No	175,313 (65.33)	400 (70.42)	175,275 (65.32)	438 (74.87)
	Yes	15,397 (5.74)	64 (11.27)	15,397 (5.74)	64 (10.94)
Diseases of the ear and mastoid process (H60–H95)	No	167,916 (62.57)	417 (73.42)	167,885 (62.57)	448 (76.58)
	Yes	22,794 (8.49)	47 (8.27)	22,787 (8.49)	54 (9.23)
Circulatory system diseases (I00–I99)	No	182,213 (67.90)	291 (51.23)	182,181 (67.89)	323 (55.21)
	Yes	8497 (3.17)	173 (30.46)	8491 (3.16)	179 (30.60)
Respiratory diseases (J00–J99)	No	121,095 (45.13)	174 (30.63)	121,026 (45.10)	243 (41.54)
	Yes	69,615 (25.94)	290 (51.06)	69,646 (25.96)	259 (44.27)
Digestive diseases (K00–K95)	No	155,825 (58.07)	189 (33.27)	155,819 (58.07)	195 (33.33)
	Yes	34,885 (13.00)	275 (48.42)	34,853 (12.99)	307 (52.48)
Skin and subcutaneous diseases (L00–L99)	No	171,697 (63.98)	335 (58.98)	171,705 (63.99)	327 (55.90)
	Yes	19,013 (7.09)	129 (22.71)	18,967 (7.07)	175 (29.91)
Musculoskeletal and connective tissue diseases (M00–M99)	No	168,726 (62.88)	305 (53.70)	168,687 (62.86)	344 (58.80)
	Yes	21,984 (8.19)	159 (27.99)	21,985 (8.19)	158 (27.01)
Genitourinary diseases (N00–N99)	No	172,532 (64.29)	293 (51.58)	172,498 (64.29)	327 (55.90)
	Yes	18,178 (6.77)	171 (30.11)	18,174 (6.77)	175 (29.91)
Pregnancy and related conditions (O00–O9A)	No	190,182 (70.87)	461 (81.16)	190,145 (70.86)	498 (85.13)
	Yes	528 (0.20)	3 (0.53)	527 (0.20)	4 (0.68)
Conditions from perinatal period (P00–P96)	No	180,648 (67.32)	379 (66.73)	180,599 (67.30)	428 (73.16)
	Yes	10,062 (3.75)	85 (14.96)	10,073 (3.75)	74 (12.65)
Congenital malformations, deformations, and chromosomal abnormalities (Q00–Q99)	No	175,930 (65.56)	298 (52.46)	175,891 (65.55)	337 (57.61)
	Yes	14,780 (5.51)	166 (29.23)	14,781 (5.51)	165 (28.21)
Injury and poisoning (S00–T88)	No	139,493 (51.98)	251 (44.19)	139,529 (52.00)	215 (36.75)
	Yes	51,217 (19.09)	213 (37.50)	51,143 (19.06)	287 (49.06)

The data collected were randomly split into a training set (50%), a validation set (15%), and a test set (35%). We chose a greater percentage of data for the test set than the validation set to ensure that we can estimate model performance with higher statistical confidence. Extreme gradient boosting (an implementation of stochastic gradient boosting) was selected for development of the machine-learning algorithm due to its ability to model complex nonlinear systems with the automated management of missing data^15^. The seminar paper on stochastic gradient boosting by Friedman (2002) described the process succinctly as follows: “Gradient boosting constructs additive regression models by sequentially fitting a simple parameterized function (base learner) to current ‘pseudo’-residuals by least squares at each iteration. The pseudo-residuals are the gradient of the loss functional being minimized, with respect to the model values at each training data point evaluated at the current step”^16^. Ten-fold cross-validation was performed to select the optimal hyper-parameters from the parameter grid space (Table 3). Parameter values that improve learning on imbalanced datasets were selected to account for the rarity of pediatric deaths and severe sepsis in the dataset, while preserving the ability of the classifier to generate true probability values. Parameter space was designed to explore multiple interaction depths that controlled model complexity. Learning rate values were selected to control the rate convergence of the algorithm, and values for the minimum reduction in loss required to make an additional partition on the leaf node of a tree were specified. The minimum sum of instance weight needed in a child node, a subsample ratio of the training instances, a subsample ratio of columns/predictors when constructing a tree, and the maximum delta step allowed for each leaf output were also considered during hyper-parameter tuning^15^. When fitting the final model, the validation set was used to determine the optimal number of trees (or boosting iterations). The test set was used in evaluating unbiased model performances^17^, alert thresholds, confusion matrices^17^, and implementation strategies for the model.

**Table 3:** Alert thresholds and model performance.

Event	Probability threshold	Sensitivity (%)	Specificity (%)	PPV (%)	NPV (%)	Relative risk	NNE
Death	0.0056	66.67	99.5	6.9	99.98	371	15
Severe sepsis	0.0028	84.5	99.00	6.31	99.99	506	16
Sepsis	0.1089	44.14	99.9	47.73	99.88	415	2
Bacteremia	0.0875	57.08	99.9	62.3	99.88	503	2

The relative importances of various predictors were measured using the “Gain”, which is a measure of the improvement in predictive power brought by a feature/variable to a given tree branch^15,18^. The Shapley Additive Explanation (SHAP) values^19,20^, a concept from game theory, was used to provide simplified inferences on how the variables/features in the model contribute to the risk of severe sepsis. The higher the SHAP value, the higher the contribution to the risk of severe sepsis. It should be noted that the simplified inference from the SHAP value does not indicate how the value of a vital sign is modified by age, gender, or other pertinent variables even though the corresponding effects have been modeled. We provide the area under the receiver operator characteristic curve (AUROC), which highlights model performance in terms of the sensitivity and specificity of the model; and the area under the precision-recall curve (AUCPR), which highlights performance in terms of the sensitivity and positive predictive values of the model.

In multiclass prediction problems, the class with the highest probability is often selected as the actual model predicted class. This approach is likely to result in sub-optimal performance in a prediction problem involving class imbalance, as is encountered when predicting the most severe measures of decompensation: death or severe sepsis. As a result, a novel stepwise multiclass classification strategy was developed to determine how patients will be classified by the model based on the predicted probabilities for each class of the outcome variable. First, each class of the outcome variable was treated as a one-versus-all system such that the sensitivity, specificity, positive predictive value (PPV), negative predictive value (NPV), relative risk, and the number needed to evaluate (NNE) can be estimated from the corresponding binary classification sub-problem. This way, per-class predicted probability thresholds can be selected to minimize per-class as well as overall model misclassification rates while preserving sensitivity on the rarest outcomes. The study team (including emergency medicine and other providers) selected levels of specificity for each class to minimize the error produced during positive classification for the class^21,22^, while maintaining acceptable levels of model sensitivity. A prioritization of the classes of the outcome variable was specified by the providers based on the perceived relative clinical significance of each class. The order of perceived clinical significance, in decreasing order, was established as severe sepsis, death, non-severe sepsis, and bacteremia. Severe sepsis was ranked as having higher clinical significance because of the expectation that patients who are indeed close to death are less likely to be missed by providers during triage than patients likely to have severe sepsis—and both are the most severe measures of decompensation in the model/study.

The study was carried out using the Cerner HealtheDataLab^23^ platform as well as the R Statistical Programming Language. Several R packages were used for the development of the machine learning model as well as estimating model performance and simplified inference^15,18,24^.

This study was approved by the Institutional Review Board of Children’s Hospital of Orange County, Orange, CA 92868 with Institutional Review Board approval number 180857. The need for informed consent was waived by the Institutional Review Board of Children’s Hospital of Orange County, Orange, CA 92868 and all aspect of the work were carried out in accordance with relevant guidelines/regulations including the Helsinki Declaration.

## Results

There were 537,837 qualifying encounters: 213 patients died; 295 had severe sepsis; 1102 had non-severe sepsis; 1103 had bacteremia without sepsis. The mortality rate, incidence of severe sepsis, incidence of non-severe sepsis, and bacteremia were 0.04%, 0.05%, 0.20%, and 0.21% respectively. Among patients with severe sepsis, 20 (6.8%) died. A total of 2048 encounters were associated with at least one of these undesirable events and markers for potential decompensation. The median and interquartile range of the ages of the patients in the study were 4 and 8 years, respectively. There were 46·4% female patients and 62.4% Hispanic patients. Summary statistics of all 46 variables considered by mortality, severe sepsis, non-severe sepsis, and bacteremia without sepsis are shown in Tables 1 and 2. T-tests and Chi-squared tests were carried out to assess univariable (unadjusted) tests of association at an alpha level of 0.05. Firstly, all variables were associated with mortality except viral vaccines, body temperature, history of conditions affecting the ear and mastoid process, history of pregnancy or related conditions, and patient sex. Secondly, all variables were associated with severe sepsis except the use of supplemental oxygen device, history of conditions affecting the ear and mastoid process, and patient ethnicity. Thirdly, only history of conditions affecting the ear and mastoid process, history of pregnancy or related conditions, ethnicity, and patient sex did not achieve univariable significance with non-severe sepsis. Lastly, only history of conditions affecting the ear and mastoid process were not significant in relation to bacteremia. The large number of variables with univariable significance in the data may have been partly driven by the large sample size in this study.

**Figure 1:**
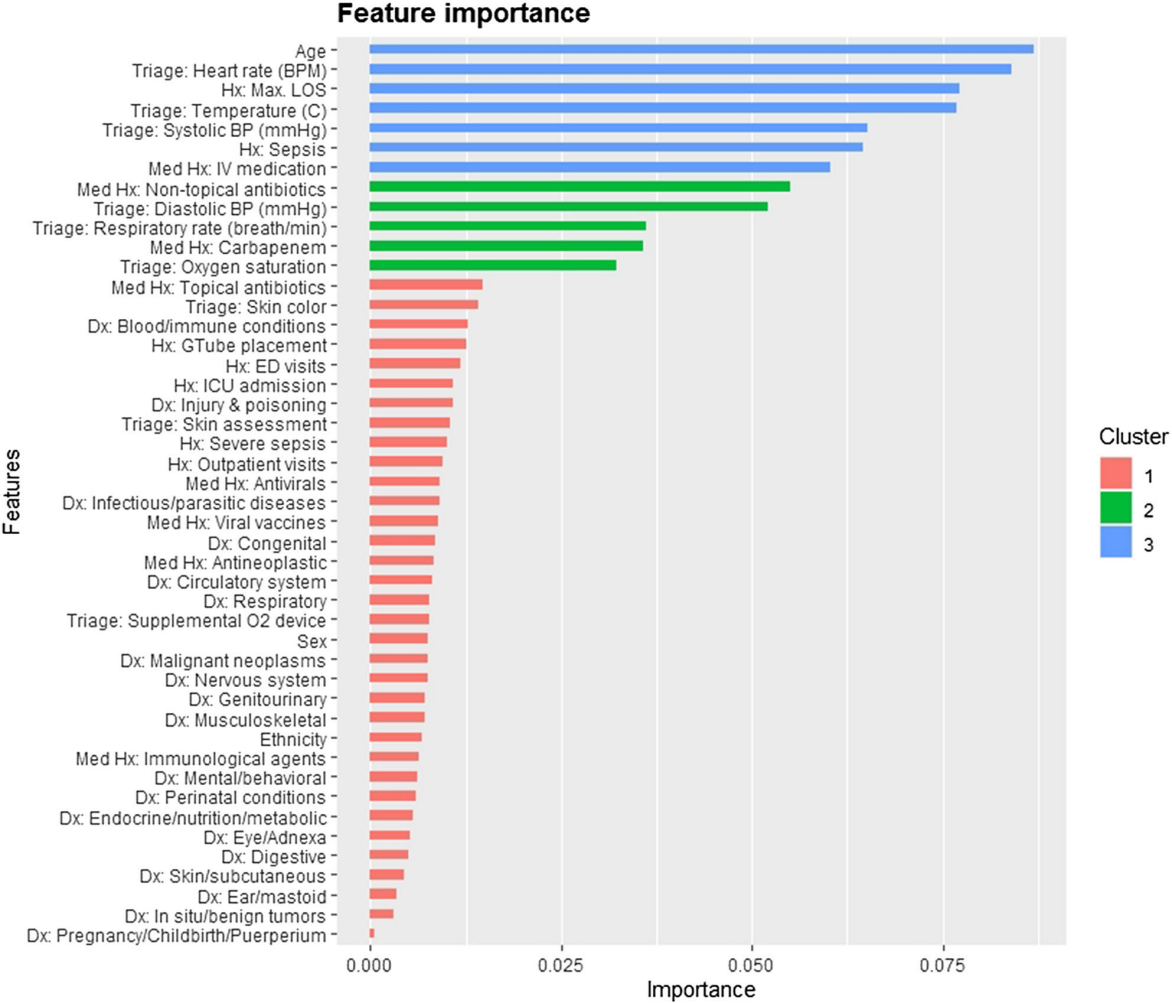
Feature/variable importance.

**Figure 2:**
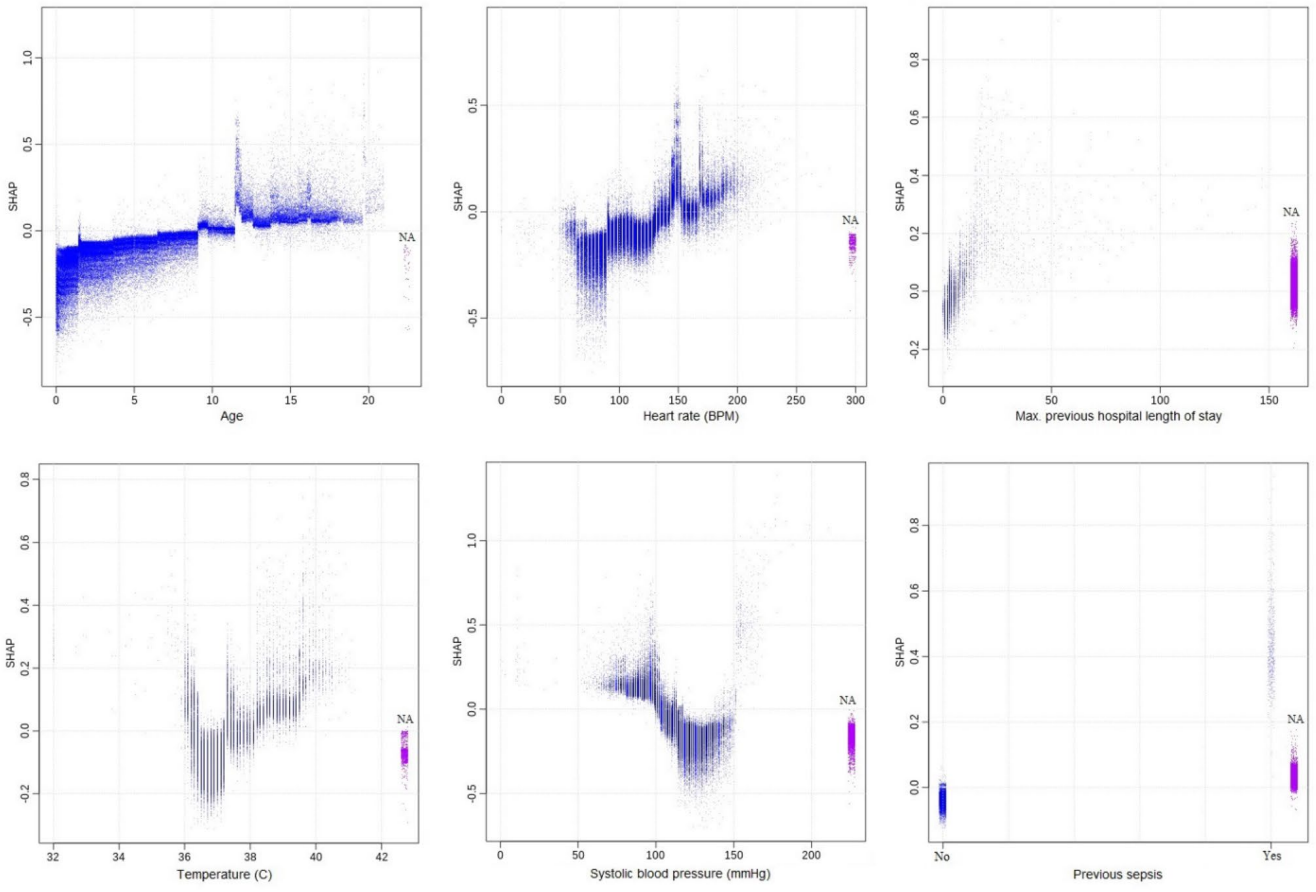
Shapley additive explanation for severe sepsis: top 6 most important features.

**Figure 3:**
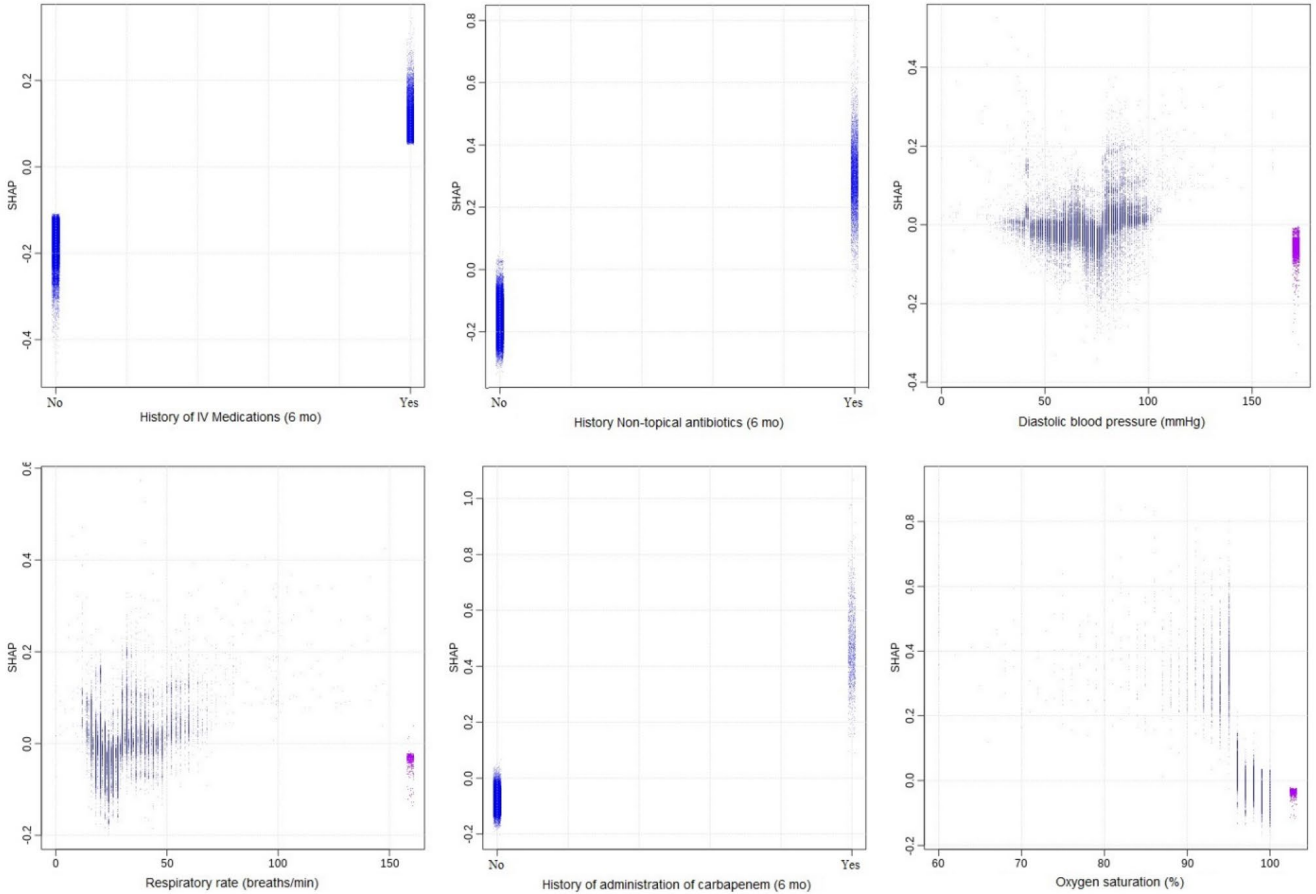
Shapley additive explanations for severe sepsis: second 6 most important features.

In Fig. 1, we show the feature importance of the variables/features in the model in decreasing order. The gradient boosting model consisted of 31 boosting iterations/trees, maximum tree depth of 8, learning rate of 0.3, and maximum delta step of 8. SHAP plots and simplified explanations of the clinical presentations that inflate the risk of severe sepsis for the top 12 most important variables are shown in Figs. 2 and 3.

The most important feature for predicting decompensation (mortality, severe sepsis, non-severe sepsis, and bacteremia) on ED triage was age. Older children are at higher risk of severe sepsis than their younger peers. The importance of age may lie in how the feature modifies others, such as vital signs. As expected, patients with abnormal vital signs were found to be at the highest risk for sepsis, as shown in Figs. 2 and 3. These figures were obtained by fixing the values of other variables at the average value for continuous variables and the most common categorical levels for categorical variables. This is a simplification of the nonlinear cross-dependences captured by the model between each vital sign and other vital signs and variables included in the model. This nonlinear cross-dependence is likely captured with a maximum tree depth of 8, indicating up to 7-way nonlinear interactions between variables in the model. This implies that the effect of a fever (or high heart rate etc.) on the risk of severe sepsis has been considered while factoring the age and sex of the patient as well as the values of other vital signs and variables in the model. This is the strength that machine learning models bring to the modeling of complex medical conditions or events.

We included some novel features/variables in the model that were among the 12 most important features. These include histories of the maximum value of previous hospital length of stay, diagnosis of sepsis, intravenous medications, non-topical antibiotics, and carbapenems. Averaged over the effect and interactions with other variables, there is increased risk for severe or sepsis-related decompensation among patients with history of longer hospital length of stay, sepsis, non-topical antibiotics, and carbapenems.

### Model performance and risk stratification

**Figure 4:**
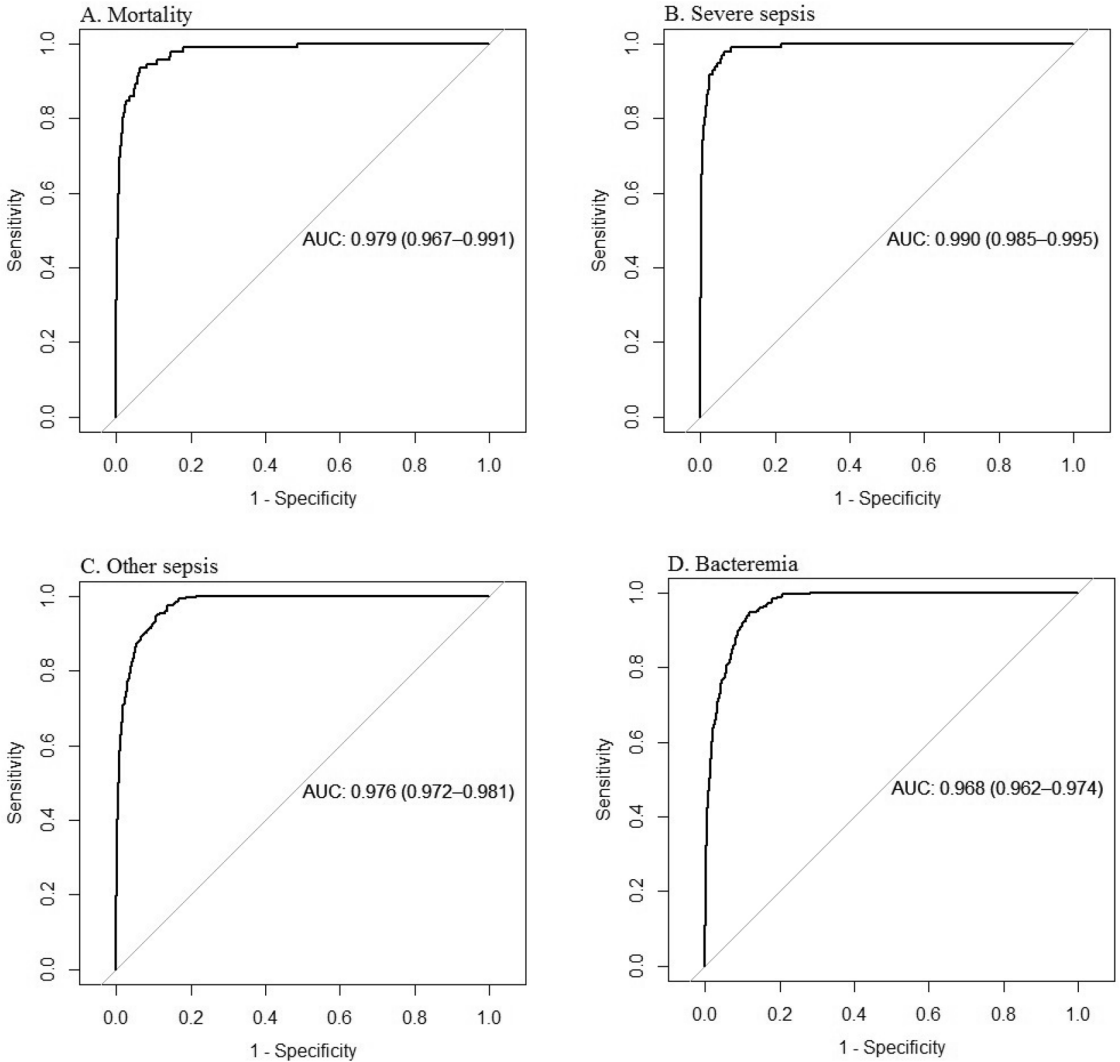
(**a**) One-versus-all areas under the receiver operator characteristic curves for mortality. (**b**) One-versus-all areas under the receiver operator characteristic curves for severe sepsis. (**c**) One-versus-all areas under the receiver operator characteristic curves for other/non-severe sepsis. (**d**) One-versus-all areas under the receiver operator characteristic curves for bacteremia.

**Figure 5:**
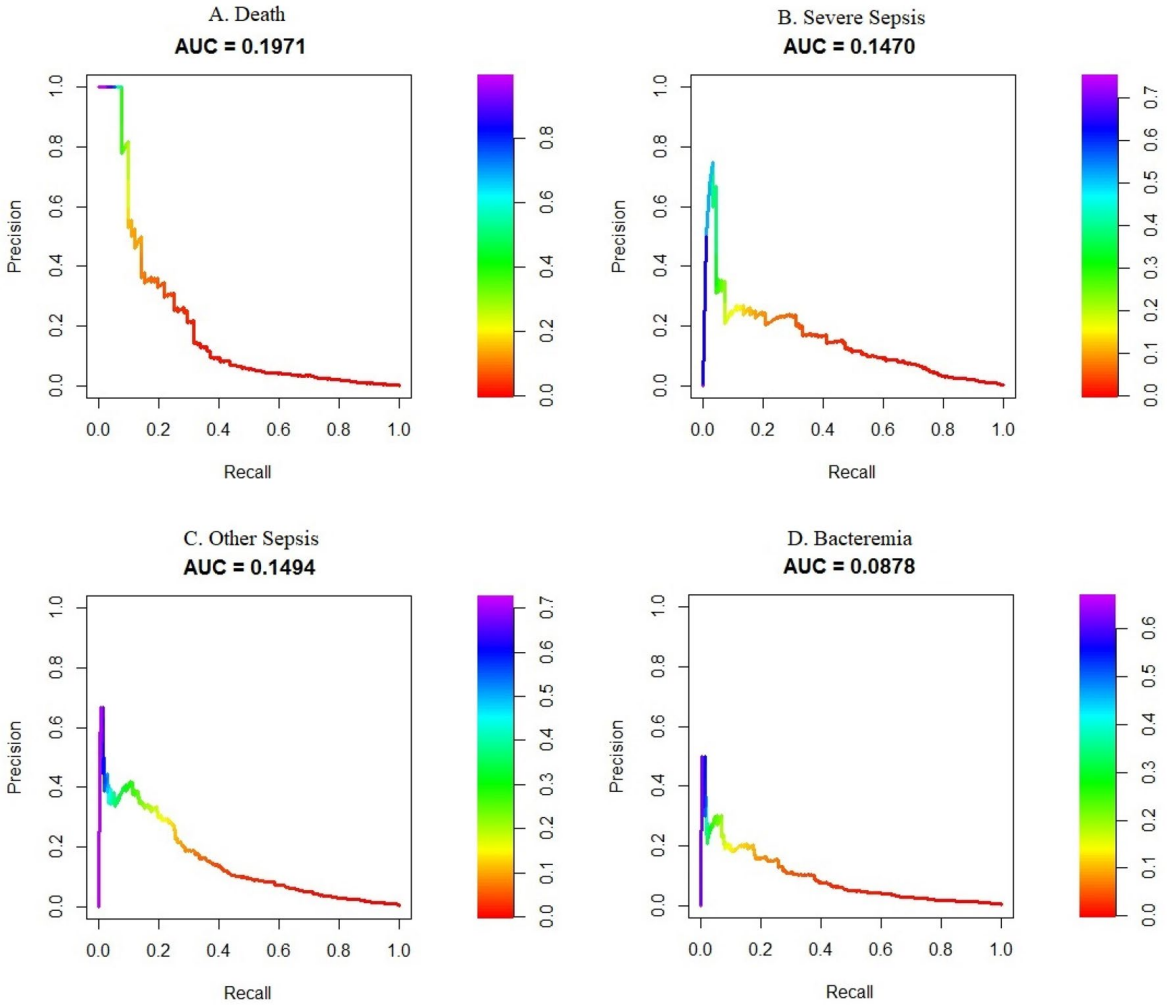
(**a**) One-vs-all areas under the precision-recall curves for mortality. (**b**) One-vs-all areas under the precision-recall curves for severe sepsis. (**c**) One-vs-all areas under the precision-recall curves for other/non-severe sepsis. (**d**) One-vs-all areas under the precision-recall curves for bacteremia.

The per-class one-versus-all AUROC mortality, severe sepsis, non-severe sepsis, and bacteremia were 0.979 (0.967, 0.991), 0.990 (0.985, 0.995), 0.976 (0.972, 0.981), and 0.968 (0.962, 0.974), respectively—see Fig. 4. The corresponding multi-class macro average AUROC and AUCPR were 0.977 and 0.316, respectively. The one-vs-all AUCPRs are provided in Fig. 5.

We used a stepwise approach for class selection that differs from simply choosing the output class with the highest predicted probability from the softmax (multi-class) output. We first selected a threshold for classifying a patient at high risk of severe sepsis using model performance statistics from a one-vs.-all approach to address the problem of class imbalance and ensure that model sensitivity is not lost to model specificity. Given the extreme class imbalance, we selected a specificity of 99.5%, resulting in a predicted probability threshold for severe sepsis of 0.0066. The resulting performance for predicting severe sepsis included sensitivity of 71.1% (62.1%, 80.2%), positive predictive value of 6.8% (5.3%, 8.4%), negative predictive value of 99.99% (99.98%, 99.99%), relative risk of 457.3 (296.0, 706.3), and an F-1 score of 0.125. In the same way, we fixed the specificity for mortality at 99.5% in a one-vs-all approach. The resulting performance for predicting mortality included sensitivity of 54.3% (43.0%, 63.5%), positive predictive value of 5.0% (3.6%, 6.3%), negative predictive value of 99.98% (99.97%, 99.98%), relative risk of 215.8 (143.9, 323.4), and F-1 score of 0.091. For the remaining 3 classes, we chose the class with the highest predicted probability if the conditions for flagging severe sepsis and death were not met. This approach resulted in balanced accuracy^25,26^ in predicting severe sepsis ranging from 52 to 85%. In a similar way, the balanced accuracy^25,26^ for predicting mortality increased from 54 to 62% using this modified approach.

The AUROC overestimates the model performance while the AUCPR underestimates it. In Table 3, we provide an unbiased statistics to help better assess the model. The potential for alert fatigue at an acceptable level of sensitivity gives us more concrete and assessible way of evaluating the model. One patient in 15 flagged at risk for death will die with sensitivity of 67% (not counting patients with severe sepsis who later die). One patient in 16 flagged at risk for severe sepsis will develop severe sepsis with sensitivity of 85%. One in two patients flagged at risk for other (non-severe) sepsis or bacteremia will suffer the respective outcome. False positives for death and severe sepsis are also of interest to ED providers because they bear similarity to patients who die or had severe sepsis or may be suffering other forms of deterioration.

## Discussion

Severe sepsis is a life-threatening response to infection and is a leading cause of death in children, with 75,000 cases resulting in 6500 deaths and a nearly $5 billion burden of care per year in the U.S. alone as well as a morbidity of 8–21%^1–4^. Early diagnosis and intervention significantly improves outcomes, however severe sepsis in children remains difficult to identify. Instances of severe sepsis continue to rise, and current rule-based models are insufficient in predicting its onset.

Accurate patient assessments within seconds of triage in the ED would allow clinicians to effectively manage patient decompensation, thus improving clinical outcomes and ultimately increasing the cost-effectiveness of the care administered. An effective algorithm would differentiate risk for critical events such as death and severe sepsis from risk for less critical events such as non-severe sepsis and bacteremia (without sepsis).

Several inpatient surveillance tools and rules-based systems such as the SIRS criteria and qSOFA are currently in widespread use^5,9,27,28^. In a 2017 study that compared the utility of the SIRS criteria and the qSOFA score, it was noted that the mean time from arrival at the ED to the identification of risk for decompensation was 47.1 min for SIRS documentation and 84·0 min for the qSOFA^5^. Clinicians must decrease the amount of time that elapses before patients at severe risk of decompensation are identified in order to deliver the most effective care and to help mitigate complications including death. On implementation of the model described here in the EMR of the corresponding author, the time from triage to prediction was between 5 and 7 s—a significantly earlier predictor for sepsis and related decompensation.

Previous studies have investigated the ability to predict general infection, sepsis, or severe sepsis among patients in the ED, intensive care unit, or general hospital ward using vital signs^29–33^. Vital signs and laboratory test results were used as predictors. In this study, we considered vital sign measurements captured during triage as well as the medical history of the patient during the 6 months prior to the index visit. We used these data to develop a real-time predictive model that could classify pediatric patients under a continuum of risk for critical decompensation.

The vital sign measurements obtained from the pediatric patients seen at our ED were similar to those reported previously. The machine-learning algorithm that we developed using stochastic gradient boosting learned the thresholds and interactions with age, other vital signs, and other relevant variables such as prior diagnoses. We identified a relationship between prior resource utilization and severe sepsis and decompensation. Patients with previous hospitalizations are generally at increased risk for sepsis with this risk increasing with the length of stay of these previous visits. Our results suggest that considerations of the patient’s history of previous sepsis diagnosis and treatment with antibiotics would improve the accuracy of tools currently used to screen for sepsis. Risk of severe sepsis is elevated in patients who had received carbapenem and other antibiotics during a previous hospitalization. This may indicate that there are lingering effects of past or recent bacterial infections treated with carbapenem that may elevate the risk of developing severe sepsis. This also suggests that certain patients are more susceptible to severe complications from infections. Identifying these high-risk patients in a timely manner can improve patient outcomes.

The model performance estimates were relatively good given the class imbalance (patients with sepsis compared to those without). But the relatively high number of false positives required to capture a true positive (as captured by the number needed to evaluate) would likely run the risk of alert fatigue for severe sepsis if the positive predictive values are not properly communicated to providers. However, further analyses of the false-positive predictions in the individual strata revealed that these groups of patients had high morbidity even without the diagnosis of severe sepsis. These false-positive patients included those requiring hospitalization and admission to the intensive care unit (ICU). In other words, even the model’s false positives for severe sepsis are of clinical utility to providers in the ED in predicting the risk of other decompensation events. This raises the question of whether the subject of deterioration is possibly more important to clinicians than just identification of sepsis or severe sepsis since screening and identification of criticality, of any kind, could be helpful to stimulate early and more general interventions. We believe that specific identification of sepsis and severe sepsis can prompt specific goal-directed interventions such as improved door to antibiotic times and early fluid resuscitation both of which are believed to be clinically important. Further, alerts generated on individual disease screening could be more useful than general alerts of criticality when it comes to achieving provider compliance with order sets.

This study had several limitations. Sepsis was identified using diagnosis codes and the corresponding model was created using a single center dataset. Additional studies based on the use of a larger multicenter dataset could provide a more accurate model. Although previous studies have demonstrated the utility of laboratory examinations and physician documentation for early sepsis identification^34^, we did not consider laboratory test values because laboratory results are not available at the time of triage. Another potential limitation, inherent in the use of electronic applications that depend on data, is the impact of bad or unexpected data. Data hastily entered during patient registration and during ED triage may invariably include errors that could result in unexpected failure, underestimation, or overestimation of risk. Also, patients who are likely to have sepsis, proceed to severe sepsis, and die will be captured in only one category. As a result, all 3 corresponding predictions should be taken seriously.

The performance of our model indicates that a high proportion of patients with severe sepsis will be captured by the model. Notably, even false-positive predictions are of clinical value. The quality of care of patients at risk of severe sepsis can be improved if the predictions of the model are used in tandem with intervention protocols for severe sepsis. It is well known that early antibiotics and fluid resuscitation can improve clinical outcomes and prevent death in patients with severe sepsis^7^. Early identification of this subset of patients will lead to better care. The inpatient ward can be notified much earlier regarding the transfer of a high-risk patient. This could improve the early allocation of resources for such hospitalizations as well as increase the amount of time available for the clinical team to prepare for these patients, who are likely to require intensive care and/or close monitoring. The stochastic gradient boosting algorithm, extreme gradient boosting, was selected for this prediction tasks because it can appropriately handle missing data without convoluted statistical imputation processes, and it is very competitive with other algorithms in terms of model performance. This model can be implemented electronically and automatically integrated within the electronic medical record to provide an early warning tool for patients at risk of severe sepsis and poor outcomes within a few seconds of ED triage. In conclusion, the development of an extreme gradient boosting model based on selected ED triage variables can be used as an early warning tool for severe sepsis.

## Supplementary Information

Supplementary Information.

## Data Availability

The dataset analyzed in this study are available from the corresponding authors on reasonable request and upon approval by the Institutional Review Board (IRB) of the corresponding authors’ institution to share the data.
