# Supplementary material for: Development and validation of an early warning tool for sepsis and decompensation in children during emergency department triage
Source: Sci Rep. 2021 Apr 21;11:8578. doi: 10.1038/s41598-021-87595-z (PMC8060307; doi:10.1038/s41598-021-87595-z)
Supplement: Supplementary file 1 — Supplementary Information. [file 41598_2021_87595_MOESM1_ESM.docx]

**Supplemental Material: Sepsis identification using diagnosis codes**

|  |  |  |
| --- | --- | --- |
| **Diagnosis code** | **Diagnosis description** | **Diagnosis code type** |
| 003.1 | Salmonella septicemia | ICD-9-CM |
| 038 | Streptococcal septicemia | ICD-9-CM |
| 038.1 | Staphylococcal Septicemia | ICD-9-CM |
| 038.1 | Staphylococcal septicemia, unspecified | ICD-9-CM |
| 038.11 | Methicillin susceptible Staphylococcus aureus septicemia | ICD-9-CM |
| 038.12 | Methicillin resistant Staphylococcus aureus septicemia | ICD-9-CM |
| 038.19 | Other staphylococcal septicemia | ICD-9-CM |
| 038.2 | Pneumococcal septicemia [Streptococcus pneumoniae septicemia] | ICD-9-CM |
| 038.3 | Septicemia due to anaerobes | ICD-9-CM |
| 038.4 | Septicemia due to gram-negative organism, unspecified | ICD-9-CM |
| 038.41 | Septicemia due to hemophilus influenzae [H. influenzae] | ICD-9-CM |
| 038.42 | Septicemia due to escherichia coli [E. coli] | ICD-9-CM |
| 038.43 | Septicemia due to pseudomonas | ICD-9-CM |
| 038.44 | Septicemia due to serratia | ICD-9-CM |
| 038.49 | Other septicemia due to gram-negative organisms | ICD-9-CM |
| 038.8 | Other specified septicemias | ICD-9-CM |
| 038.9 | Unspecified septicemia | ICD-9-CM |
| 054.5 | Herpetic septicemia | ICD-9-CM |
| 670.22 | Puerperal sepsis, delivered, with mention of postpartum complication | ICD-9-CM |
| 771.81 | Septicemia [sepsis] of newborn | ICD-9-CM |
| 995.91 | Sepsis | ICD-9-CM |
| A02.1 | Salmonella sepsis | ICD-10-CM |
| A20.7 | Septicemic plague | ICD-10-CM |
| A21.7 | Septicemic plague | ICD-10-CM |
| A22.7 | Anthrax sepsis | ICD-10-CM |
| A24.1 | Acute and fulminating melioidosis (melioidosis sepsis) | ICD-10-CM |
| A26.7 | Erysipelothrix sepsis | ICD-10-CM |
| A32.7 | Listerial sepsis | ICD-10-CM |
| A39.2 | Acute meningococcemia | ICD-10-CM |
| A39.3 | Chronic meningococcemia | ICD-10-CM |
| A39.4 | Meningococcemia, unspecified | ICD-10-CM |
| A40.0 | Sepsis due to streptococcus, group A | ICD-10-CM |
| A40.1 | Sepsis due to streptococcus, group B | ICD-10-CM |
| A40.3 | Sepsis due to Streptococcus pneumoniae | ICD-10-CM |
| A40.8 | Other streptococcal sepsis | ICD-10-CM |
| A40.9 | Streptococcal sepsis, unspecified | ICD-10-CM |
| A41.01 | Sepsis due to Methicillin susceptible Staphylococcus aureus | ICD-10-CM |
| A41.02 | Sepsis due to Methicillin resistant Staphylococcus aureus | ICD-10-CM |
| A41.1 | Sepsis due to other specified staphylococcus | ICD-10-CM |
| A41.2 | Sepsis due to unspecified staphylococcus | ICD-10-CM |
| A41.3 | Sepsis due to Hemophilus influenzae | ICD-10-CM |
| A41.4 | Sepsis due to anaerobes | ICD-10-CM |
| A41.50 | Gram-negative sepsis, unspecified | ICD-10-CM |
| A41.51 | Sepsis due to Escherichia coli [E. coli] | ICD-10-CM |
| A41.52 | Sepsis due to Pseudomonas | ICD-10-CM |
| A41.53 | Sepsis due to Serratia | ICD-10-CM |
| A41.59 | Other Gram-negative sepsis | ICD-10-CM |
| A41.81 | Sepsis due to Enterococcus | ICD-10-CM |
| A41.89 | Other specified sepsis | ICD-10-CM |
| A41.9 | Sepsis, unspecified organism | ICD-10-CM |
| A42.7 | Actinomycotic sepsis | ICD-10-CM |
| B37.7 | Candidal sepsis | ICD-10-CM |
| O03.37 | Sepsis following incomplete spontaneous abortion | ICD-10-CM |
| O03.87 | Sepsis following complete or unspecified spontaneous abortion | ICD-10-CM |
| O85 | Puerperal sepsis | ICD-10-CM |
| P36.0 | Sepsis of newborn due to streptococcus, group B | ICD-10-CM |
| P36.10 | Sepsis of newborn due to unspecified streptococci | ICD-10-CM |
| P36.19 | Sepsis of newborn due to other streptococci | ICD-10-CM |
| P36.2 | Sepsis of newborn due to Staphylococcus aureus | ICD-10-CM |
| P36.30 | Sepsis of newborn due to unspecified staphylococci | ICD-10-CM |
| P36.39 | Sepsis of newborn due to other staphylococci | ICD-10-CM |
| P36.4 | Sepsis of newborn due to Escherichia coli | ICD-10-CM |
| P36.8 | Other bacterial sepsis of newborn | ICD-10-CM |
| P36.9 | Bacterial sepsis of newborn, unspecified | ICD-10-CM |
| T81.44XA | Sepsis following a procedure, initial encounter | ICD-10-CM |
